# Using theory of change to design and evaluate public health interventions: a systematic review

**DOI:** 10.1186/s13012-016-0422-6

**Published:** 2016-05-06

**Authors:** Erica Breuer, Lucy Lee, Mary De Silva, Crick Lund

**Affiliations:** 1 Alan J. Flisher Centre for Public Mental Health, Department of Psychiatry and Mental Health, University of Cape Town, 46 Sawkins Road, Rondebosch, 7700 Cape Town, South Africa; 2 Centre for Global Mental Health, London School of Hygiene and Tropical Medicine, Keppel Street, London, WC1E 7HT UK

**Keywords:** Theory of change, Intervention development, Evaluation, Programme theory, Public health, Systematic review

## Abstract

**Background:**

Despite the increasing popularity of the theory of change (ToC) approach, little is known about the extent to which ToC has been used in the design and evaluation of public health interventions. This review aims to determine how ToCs have been developed and used in the development and evaluation of public health interventions globally.

**Method***s***:**

We searched for papers reporting the use of “theory of change” in the development or evaluation of public health interventions in databases of peer-reviewed journal articles such as Scopus, Pubmed, PsychInfo, grey literature databases, Google and websites of development funders. We included papers of any date, language or study design. Both abstracts and full text papers were double screened. Data were extracted and narratively and quantitatively summarised.

**Results:**

A total of 62 papers were included in the review. Forty-nine (79 %) described the development of ToC, 18 (29 %) described the use of ToC in the development of the intervention and 49 (79 %) described the use of ToC in the evaluation of the intervention. Although a large number of papers were included in the review, their descriptions of the ToC development and use in intervention design and evaluation lacked detail.

**Conclusions:**

The use of the ToC approach is widespread in the public health literature. Clear reporting of the ToC process and outputs is important to strengthen the body of literature on practical application of ToC in order to develop our understanding of the benefits and advantages of using ToC. We also propose a checklist for reporting on the use of ToC to ensure transparent reporting and recommend that our checklist is used and refined by authors reporting the ToC approach.

**Electronic supplementary material:**

The online version of this article (doi:10.1186/s13012-016-0422-6) contains supplementary material, which is available to authorized users.

## Background

Most public health interventions are inherently complex, with multiple interacting components, delivered at multiple levels. This complexity makes them difficult to evaluate using traditional experimental designs. Public health interventions often rely on ongoing quality improvement based on the implementation experience. Therefore, they may not reach the level of stability required to conduct evaluations such as randomised controlled trials (RCTs) [1]. Some researchers propose that understanding the public health intervention’s underlying theory of change (ToC) and its related uncertainties may improve the evaluation of complex health interventions [1–3].

Theory-driven evaluation is a collection of evaluation methods which emphasise the importance of understanding how and why a programme works in order to evaluate it [4, 5]. By programme, we mean a set of organised activities or interventions supported by resources designed to achieve a specific result [6]. The theories are first made explicit and then used to see how the programme theory results in the intended outcomes [4]. There are several overlapping types of theory-driven evaluation approaches including logic models, logical frameworks, outcomes hierarchies, realist evaluation, and ToC [4, 5].

ToC was developed by Weiss and others [7] within the tradition of theory-driven evaluation. Although definitions of ToC vary, we define it as an approach which describes how a programme brings about specific long-term outcomes through a logical sequence of intermediate outcomes [8]. The ToC is often developed using a backward mapping approach which starts with the *long-term outcome* and then maps the required *process of chang*e and the short- and medium-term outcomes required to achieve this [9]. During this process, the *assumptions* about what needs to be in place for the ToC to occur are made explicit as well as the *contextual* factors which influence the ToC. Additional elements of a ToC can include *beneficiaries*, *research evidence supporting the ToC*, *actors in the context*, *sphere of influence*, *strategic choices and interventions*, *timelines* and *indicators* [8]. These elements are usually presented in a diagram and/or narrative summary [8].

The ToC is usually developed in consultation with stakeholders through workshops or interviews although the participation of stakeholders can vary substantially in practice [10]. For example, some ToCs are developed through a series of workshops and meetings with a wide range of stakeholders including service users [11, 12] whereas others are developed by evaluators and funders using programme documentation [13, 14]. The resulting ToCs can be used as a framework for programme development and evaluation [8]. The ToC approach is method neutral and as such does not prescribe specific types of evaluation methods such as qualitative interviews or RCTs [15].

ToC is distinct from sociological or psychological theories which describe why change occurs although these may be used to inform the ToC [3]. For example, Bauer used an ecological model of community organising to inform a ToC for a capacity and advocacy initiative for residents to impact on public health policy and training of public health professionals [16].

ToC differs from other theory-driven approaches to evaluation despite similar origins. For example, although logic models outline the inputs, processes, outputs and outcomes of a programme in a similar manner to ToC, they can be rigid and do not make explicit the causal pathways through which change happens in the way that ToC does [3]. Similarly, although logframes were initially developed to summarise discussions with stakeholders, funder-driven formats have largely reduced logframes to a results-based management tool [17]. Realist evaluation, on the other hand, comes from a perspective of scientific realism and focuses predominantly on the interaction between the context, mechanisms and outcomes of the programme. Usually used post hoc, evaluators seek to uncover the underlying programme theories. These theories are often more abstract than the theories developed through ToC or logic models [18]. The development of ToC has been influenced by Freirean thinking on how to create social change by empowering individuals [19]. Despite some fundamental differences in their theoretical underpinnings, many of these approaches are used interchangeably or together [18, 20].

ToC has been used widely in the development sector for programme development and evaluation by funders such as the UK’s Department for International Development, Comic Relief, Grand Challenges Canada and the Gates Foundation [3, 19]. However, there has been no global systematic review to our knowledge on the use of ToC for the design and evaluation of public health interventions. Coryn et al. [4] conducted a review of theory-driven evaluation more broadly. They found 45 examples of theory-driven evaluation in the peer-reviewed literature between 1990 and 2009. These evaluations included education, crime and safety and transportation interventions. Roughly half (21/45) were evaluations of health interventions [4]. A rapid analysis of the included papers in preparation for this review indicated that only three of these used ToC.

The lack of a systematic review means that there is no clear idea of how the ToC has been used and reported in the peer-reviewed and grey literature in relation to public health interventions. Given the increasing popularity of the ToC approach, understanding how it is has been used and described previously allows future users of the approach to learn from the work of others and build upon it. It also helps to move towards a more consistent way of using the ToC approach.

In this review, we sought to review both peer-reviewed and grey literature to determine how ToCs have been developed and used in the development and evaluation of public health interventions globally. Specifically, we sought to answer the following questions:

1. How are ToCs for public health interventions developed and refined?
2. How is the ToC approach used in the
  - development of an intervention;
  - implementation of the intervention;
  - development of indicators for measurement;
  - evaluation of the intervention, including statistical approaches; and
  - conceptualisation/evaluation of the influence of context.

## Methods

The authors developed a protocol for this review which was agreed prior to the commencement of the study. This is available in Web Additional file 1.

### Inclusion and exclusion criteria

The inclusion and exclusion criteria are listed in Table 1. In summary, we included studies of public health interventions which were defined as interventions which are intended to protect health or prevent or treat ill health in communities or populations [21]. We included papers describing interventions addressing any health issue in all populations which (a) described how a ToC approach was used to design, implement or evaluate a public health intervention or (b) described the development of a ToC for a public health intervention. Evaluation study designs included longitudinal studies, quantitative surveys, case study research [22] and qualitative studies.

**Table 1:** Inclusion and exclusion criteria

Inclusion criteria: • Describes or evaluates a public health intervention defined as any intervention which is intended to protect health or prevent or treat ill health in communities or populations [1] • Self-identifies as using a ToC approach and specifically mentions “theory of change” • Describes how a ToC was developed or how ToC was used in the design, evaluation and/or implementation of a public health intervention • Any evaluation design • Any date • Any language • Any country Exclusion criteria: • Conceptual/methodological or advocacy papers unless they include an example of how a ToC was developed or how ToC was used in the design, evaluation and/or implementation of a public health intervention • Review articles • Specific psychological, sociological or organisational theory (unless used to inform the ToC) • ToC in which the outcome is a change within an individual rather than change at population level.	ᅟ

We required papers to specifically mention that they used “theory of change” and excluded those who did not for the following reasons. Firstly, as described above, there are a range of overlapping definitions for ToC and other programme evaluation methods. Given the often minimal amount of detail provided about the programme theory in papers, and especially in abstracts, it would be difficult to enforce a standard criteria for ToC against which papers could be evaluated for inclusion. Secondly, piloting the initial broad search strategy (including all synonyms for ToC and programme logic) returned more than 20,000 hits in only three databases. By refining the criteria to specify ToC by name, we were able to thoroughly explore literature which explicitly self-identified using ToC.

As the focus of this review was on public health interventions, we excluded papers in which the long-term outcome of the ToC was a change within an individual rather than change in the population. For example, a ToC describing how cognitive behavioural therapy may impact on an individual’s cognitive processes and behaviour would be a change within the individual. However, if the focus of the ToC was on how a cognitive behavioural therapy intervention impacted the prevalence of depression would be change in the population. We excluded reviews and methodological or advocacy papers unless they included an example of how a ToC was developed or how ToC was used in the design, evaluation and/or implementation of a public health intervention. We did not limit the inclusion by date, language, study design or type of publication.

### Search strategy

The database searches were conducted between the 16th November and the 4th December 2013 by EB. The main search term used was “theory of change”. Where the database allowed, we limited this to health or healthcare and to humans. We searched databases of peer-reviewed journal articles (Scopus, PubMed, PsychInfo, Science Citation Index, Social Science Citation Index, Academic Search Premier, Africa-Wide Information, CINAHL and BIOSIS). An example of a search string used for PyschInfo was “theory of change” AND (“health” OR “healthcare” OR “health services” OR “medicine”). We also searched grey literature databases (The Directory of Published Proceedings OpenGrey, Disability Archive UK, Eldis, Popline, DFID Research for Development, SciDevNet and World Bank Documents and Reports) and the first 50 pages of a Google search. The websites of Comic Relief, DFID, Grand Challenges Canada, The Bill and Melinda Gates Foundation, HIVOS, World Vision, the Robert Wood Johnson foundation, Actknowledge and the Theory of Change Community were also searched. In addition, we contacted experts in the field and sent requests for papers to two existing global mailing lists for evaluators: MandENEWS and Pelican.

### Screening and eligibility

Following the search of databases of peer-reviewed journal articles, the titles and abstracts of the search results from peer-reviewed papers were exported into Endnote [23] where duplicates and irrelevant titles were removed. The peer-reviewed journal articles found through contact with experts were added to this. The titles and abstracts were double screened by EB and LL against the inclusion and exclusion criteria. Once the abstracts were screened, the full papers or reports of the included abstracts were obtained and assessed for eligibility by both reviewers.

Following the grey literature search as described above, all potentially relevant results were saved into Evernote [24]. These were double screened by both reviewers against the inclusion and exclusion criteria.

Any differences between authors’ opinions were resolved via discussion throughout the review process.

### Data extraction and analysis

The data from the papers were extracted by the first author (EB) onto a data extraction form. This included information on authors, publication dates, the type of interventions and outcomes, the development of ToC, the use of ToC in the design, implementation and evaluation of the intervention and the influence of context. The data collection form also included key principles of theory-driven evaluation proposed by Coryn et al. [4]. These included how the programme theory was (a) formulated, (b) used to formulate and prioritise evaluation questions, (c) plan and conduct evaluations, (d) inform the measurement of constructs in the programme theory and (e) provide a causal explanation. Where a paper described or showed a ToC, we assessed what elements of ToC they presented. The list of ToC elements was adapted from Vogel and included context, long-term change, process/sequence of change and assumptions [8]. However, as there is no agreed upon assessment of quality for papers reporting ToC, we did not asses the quality of the included papers. We did not contact authors for additional information.

Descriptive statistics were calculated using STATA 13 [25]. The papers were compared, evaluated and summarised narratively in relation to review questions. Due to the heterogeneity of the study designs, interventions and outcomes included in this review, a meta-analysis was not conducted.

## Results

### Search results

**Fig. 1:**
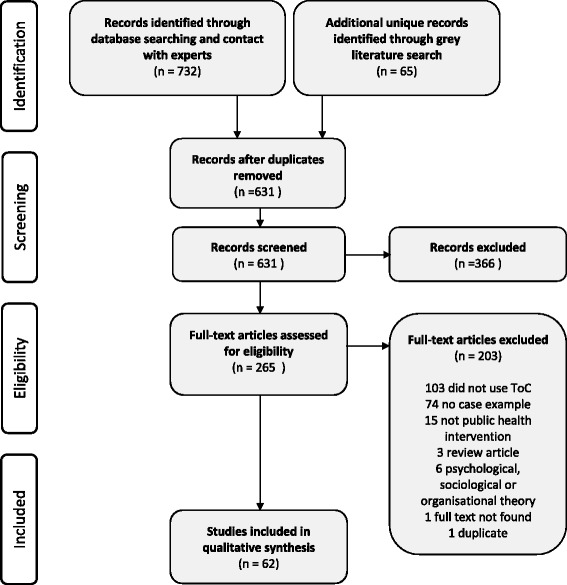
Results of database, abstract and full text screening

In total, 566 abstracts were screened, resulting in 200 full text peer-reviewed articles which were assessed for eligibility. An additional 65 records were identified from the grey literature search and screened for eligibility. A total of 62 papers were included [1, 12–14, 16, 26–82]. Figure 1 is adapted from the PRISMA guidelines [83] and summarises the search process and results.

### Included studies

**Fig. 2:**
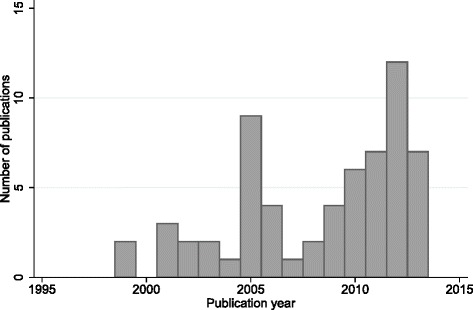
Histogram of number of publications per year

The publication dates of the papers range between 1999 and 2013, with a steady increase in papers over time (Fig. 2). The majority were published in English in peer-reviewed journals, but we also included PhD theses, presentations and NGO reports from the grey literature. Most of the research was conducted in the USA or the UK. More details are provided in Table 2. Four pairs of papers are reported on the same public health interventions [1, 13, 42, 43, 54, 60, 81, 82]. However, as the primary interest of this paper is how the use of ToC is described in reports and peer-reviewed journal articles, we have included them as separate papers.

**Table 2:** Basic descriptive statistics of included papers

Variable	*n* = 62
	*n* (%)
Language	
English	60 (96.7 %)
Spanish	2 (3.2 %)
Country	
USA	28 (45.2 %)
UK	20 (32.3 %)
Other high-income country	5 (8.1 %)
Low- and middle-income country	9 (14.5 %)
Type of publication	
Grey literature	15 (24.2 %)
Peer-reviewed journal article	47 (75.8 %)
*Public health, medicine and nursing*	*31 (50.0 %)*
*Psychology*	*3 (4.8 %)*
*Social policy and social work*	*6 (9.7 %)*
*Evaluation methods*	*5 (8.1 %)*
*Other*	*2 (3.2 %)*
Use of TOC	
Describes development of ToC	49 (79.0 %)
Describes the use of ToC in the development of the intervention	18 (29.0 %)
Describes the use of ToC in the evaluation of the intervention	49 (79.0 %)

A variety of types of public health interventions reported using ToC in the design, development and evaluation of public health interventions (Table 3). These included systems of care for adolescents with behavioural and emotional difficulties [12, 26, 50, 53, 54, 56, 61, 65, 80–82], substance use interventions [27, 49], domestic violence interventions [29], comprehensive community initiatives [13, 16, 35, 62, 81], medication supply among community health workers [40] and integrated district level mental healthcare plans in low- and middle-income countries [55].

**Table 3:** Characteristics of studies included in the review and reported aspects of the ToC process

Reference	Location(s)	Brief description of program	Health outcome	Development of TOC	TOC in intervention development	ToC in evaluation
Andersen, Nesman et al. (2012) [26]	USA	Tampa Hillsborough integrated network for kids: respite care for families with seriously emotionally disturbed children	Reduction in caregiver burden	X		
Andreas, Ja et al. (2010) [27]	USA	Peer community approach to prevent substance use and recidivism in men and women in recovery who have been incarcerated	Prevention of substance abuse relapse	X		X
AusAID (2010) [28]	Papua New Guinea	Strengthen sector wide response to HIV in Papua New Guinea	Stable HIV incidence rate; improved care for people living with HIV/AIDS	X		
Bacchus, Bewley et al. (2010) [29]	UK	Guidelines, staff training, inclusion of routine enquiry for domestic violence with all patients, and referral of women disclosing violence to an on-site advocacy service.	Reduction in severity and frequency of abuse. Improved safety and access to resources	X		X
Barton, Powers et al. (2001) [30]	USA	Promoting positive youth development for young people	Reduction in drug and alcohol use, increase in immunisation rates			X
Basson and Roets (2013) [31]	South Africa	A workplace wellness programme for HIV affected nurses	Positive health and well-being of nurses	X	X	
Bauer (1999) [16]	USA	Oakland Community-Based Public Health Initiative: a capacity and advocacy initiative for residents to impact on public health policy and training of public health professionals	Nil specific	X		X
Bauld, Judge et al. (2005) [32]	UK	Health action zones: a multi-area study in 26 local health areas aiming to identify and address the public health needs of the local area, to increase effectiveness and efficiency of services and develop partnerships	Improved health and reduced inequality.			X
Beeston, Robinson et al. (2011) [33]	UK	A strategy for reducing alcohol related harm	Reduced alcohol related harms	X		X
Bhattacharjee (2013) [34]	India	A multipronged programme targeting sex workers, their partners and the community to increase condom use and reduce violence towards sex workers	Increase in protected sex and decrease in STI/HIV transmission among sex workers	X	X	X
Birkby (2001) [35]	USA	Community partnerships for protecting children initiative on child maltreatment consisting of 5 strategic elements including casework training, family decision-making, a hotline, community resource teams and substance abuse prevention and treatment	Reduction in serious injury	X		X
Bonner (2003) [36]	UK	Programme aimed at reducing drug taking and drug related harm	Reduction in drug taking and drug-related harm among urban young people			X
Brown, Hawkins et al. (2013) [37]	USA	Communities that care: a manualised system for community coalitions to influence human and financial resources to address adolescent health and behaviour problems	Reduction in adolescent behaviour problems.			X
Carr, Lhussier et al. (2008) [38]	UK	A walking group as part of the Positive Health Programme funded by the Neighbourhood Renewal Fund	Enhanced physical fitness	X		X
Carroll, David et al. (2005) [39]	UK	Workplace wellness program	Improved well-being of women clothing factory workers	X		X
Chandani, Noel et al. (2012) [40]	International multi-country	Medication supply chains for community health workers in Rwanda, Ethiopia and Malawi	Appropriate receipt of treatment for common childhood illness; Availability of usable and quality medicines for appropriate treatment of childhood illnesses	X	X	X
Cole, Hogg-Johnson et al. (2006) [41]	Canada	Workplace economic program	Reduction in pain intensity and disability			X
De La Rosa, Perry et al. (2005) [42]	USA	A family based home visit intervention during pregnancy and after the birth of the first child	Improved social support, caregiver behaviours, family interactions and decreased personal problems affecting parenting.	X	X	X
De La Rosa, Perry et al. (2009) [43]	USA	A family based home visit intervention during pregnancy and after the birth of the first child	Multiple including improved immunisation rates, connection with a medical home and maternal achievement of economic self-sufficiency.	X	X	X
Dixon-Woods, Bosk et al. (2011) [44]	USA	An intervention in intensive care units to reduce central venous catheter bloodstream infections	Decrease in intensive care unit mortality, hospital mortality, catheter related infections, ventilator associated pneumonia	X		X
Dixon-Woods, Tarrant et al. (2010) [45]	UK	Safer Patient Initiative: introduction of patient safety into hospital management, culture and practice.	Increased patient safety in hospitals	X		
Goss-Power (2005) [46]	USA	VASE: a school for adolescents with emotional and behavioural disturbances	Nil specific			X
Gray and Seddon (2005) [47]	UK	Two programmes aimed at children in “trouble” at school, truancy and risk of social exclusion. 1. “Kick it” Football Project which included mentoring and drug education, 2. The Salford Anti-Rust gardening project using mentoring using horticulture	No specific health outcomes mentioned (mechanisms for change only)	X		X
Gregor (2009) [48]	UK	Programme which enables partnership between public and third sector organisations to deliver awareness-raising programs	Earlier presentation with TB, decrease in TC incidence Decrease in TB stigma, decreased barriers to access	X		X
Henderson (2004) [49]	USA	A substance abuse treatment programme for homeless people	Sobriety, improved medical health			X
Hernandez and Hodges (2006) [12]	USA	Interagency service planning for youth who had been arrested and involved in juvenile probation	No specific health outcomes	X	X	
Illinois Caucus for Adolescent Health (2013) [50]	USA	A network of youth and adults who advocate within school, family and healthcare systems to support sexual health, rights and identities of youth	Impacts school, family and healthcare systems in priority areas	X		
Kemp, Harris et al. (2013) [51]	Australia	An ante- and post-natal home visiting program	Multiple including improved pre and postnatal maternal health and increased engagement with services	X		X
Knowlton and Phillips (2012) [14]	USA	A five-arm strategic funding model to improve food security for children in the USA	Improved US food security	X		X
Kreger, Sargent et al. (2011) [52]	USA	A network of coalitions and technical assistance programmes who use an environmental justice approach to reduce risk factors for smoking	Healthier children and environments	X	X	X
Levinson-Johnson (2012) [53]	USA	A system of care for youth with behavioural and emotional problems and their families	Various health systems level changes	X	X	X
Levison-Johnson and Wenz-Gross (2010) [54]	USA	A system of care for youth with behavioural and emotional problems and their families	Not described	X		
Lund, Tomlinson et al. (2012) [55]	International multi-country	Programme for improving mental healthcare (PRIME): district specific mental health care plans which are integrated into routine health services	Not described	X	X	X
Macfarlane, Greenhalgh et al. (2011) [56]	UK	Whole-scale transformation of stroke, kidney and sexual health services including human resource management	Various including culture of health service and quality of care and service provision	X		X
Mackenzie (2006) [57]	UK	Starting well: intensive home visiting services for families of new babies in 2 areas in Scotland	Not described	X		X
Mackenzie and Blamey (2005) [58]	UK	A multipronged heart disease prevention program	Reduced coronary heart disease	X		X
Mackenzie, Blamey et al. (2007) [59]	UK	Choose life: a national strategy to reduce suicide in Scotland	20 % reduction in suicide rates over a 10-year period			X
Mackenzie, O’Donnell et al. (2010) [1]	UK	Keep Well: a programme to identify those at risk of ill health and offered health checks and preventative services within primary and secondary care	Decreasing inequalities in cardiovascular morbidity and mortality in Scotland			X
Mackenzie, Reid et al. (2012) [60]	UK	Keep Well: the programme to identify those at risk of ill health and offered health checks and preventative services within primary and secondary care	Decreasing inequalities in cardiovascular morbidity and mortality in Scotland			
Maselli (2012) [61]	USA	A comprehensive system of care which avoids re-traumatising children and youth with severe emotional challenges	Nil specific	X	X	X
Mason (2005) [62]	UK	The Timely Tales: a community development and community arts project (part of a larger Health Action Zone Project)	None described	X		X
McQuiston, Choi-Hevel et al. (2001) [63]	USA	A culture specific programme to empower lay health advisers to promote sexual health and reduce sexually transmitted diseases	Promotion of sexual health and reduction of sexually transmitted diseases including HIV	X	X	
Mookherji and LaFond (2013) [64]	International multi-country	Africa Routine Immunisation System Essentials (ARISE): using lessons from existing immunisations programmes that have achieved solid advances in immunisation	Improved immunisation performance and equity	X		X
Morilus-Black, McCarthy et al. (2012) [65]	USA	An integrated system of care for children and families experiencing social and/or behavioural challenges	Improved care and referrals	X	X	X
Reid and Botma (2012) [66]	South Africa	A programme which aims to expand public services to children with biomedical healthcare needs related to HIV	Nil specific	X		
Riley, Byng et al. (2008) [67]	UK	The Lewisham Depression Programme: a multifaceted programme which included marketing of the program, training and a depression recognition audit	Not described	X		X
Rivera, Martorell et al. (2011) [68]	International multi-country	A master plan for the improvement of nutrition in Mesoamerica	Multiple including decreased mortality and increased maternal and child health	X	X	X
Rodriguez, Betanzos-Reyes et al. (2011) [69]	International multi-country	A multifaceted strategic plan to eliminate malaria transmission in Mesoamerica	Eliminating local transmission of malaria in Mesoamerica	X	X	
Scanlon, Beich et al. (2012) [70]	USA	Quality improvement alliance: to improve quality in the healthcare system	Improvement in key community and population health outcomes	X	X	
Schierhout, Hains et al. (2013) [71]	Australia	A continuous quality improvement programme in primary health care centres	Changes in delivery of guideline schedules services, focusing on diabetes and preventive care	X		X
Secker, Bowers et al. (2005) [72]	UK	Preretirement health advice and services for people aged 50–65 years	Nil mentioned	X		X
Smith and Barnes (2013) [73]	UK	A whole systems approach to prevention of ill health	Improved quality of life, reduced social exclusion and reduced need for acute hospital care for older people	X		X
Suarez-Balcazar (2005) [74]	USA	A community intervention to assist community members in accessing health resources through the project’s home web page and the Internet.	Nil specific			X
Tran (2009) [75]	UK	Provision of mental health advocacy delivered by a Chinese advocate with Cantonese and Mandarin skills	Nil specific	X		X
Tucker, Liao et al. (2006) [76]	USA	Community strategies driven by 40 community coalitions to eliminate disparities in racial or ethnic groups for priority health areas	Reduction in health disparities	X	X	X
Vander Stoep, Williams et al. (1999) [77]	USA	A family-centred system of care by community-based teams for youth with mental health needs	Improved level of functioning for children	X		X
Veerman, De Kemp et al. (2003) [78]	Netherlands	Families First: a home-based intervention for children with behaviour problems	Nil specific			X
von dem Knesebeck, Joksimovic et al. (2002) [79]	Germany	Systems interventions to improve local coordination of health and social care	Improved health care, health monitoring and health promotion			X
Walker and Matarese (2011) [80]	USA	A coaching, training and technical assistance model for wraparound	Nil specific	X	X	
Weitzman, Silver et al. (2002) [13]	USA	Urban Health Initiative: a citywide multi-sector planning initiative	Improved health and safety outcomes for children and youth	X		X
Weitzman, Mijanovich et al. (2009) [81]	USA	Urban Health Initiative: a citywide multi-sector planning initiative	Improved health and safety outcomes for children and youth	X		X
Wenz-Gross and DuBrino (2012) [82]	USA	A which programme aims to decrease and prevent youths with serious emotional disturbance from becoming involved in the courts	Various including increased youth functioning and behavioural adjustment	X	X	X

### Development of ToCs

Forty-nine papers (79 %) included some information on the ToC development process. Forty-three percent (*n* = 27) of the papers developed their ToCs prospectively and 19.4 % (*n* = 12) retrospectively. The remainder either developed their ToC during project replanning (*n* = 3.5 %) or did not specify when they developed their ToC (*n* = 20, 32 %).

The ToCs were developed using workshops [28, 34, 47, 48, 55, 63, 64, 72, 76] and working groups [12, 53, 54, 61, 68, 69, 82], document reviews [16, 35, 44, 56, 67, 71], interviews and discussions [16, 27, 29, 35, 40, 44, 47, 56, 57, 62, 65, 66, 73, 80], surveys [31, 67], programme observation [16, 44, 45, 56, 67], literature reviews [33, 40, 68, 69, 80] and existing conceptual frameworks or theory [33, 40, 42–44, 51, 64, 68, 69]. The ToC development included consultations or interviews with the following stakeholders: programme staff [27, 38, 40, 44, 45, 52, 54, 57, 63, 65–67, 72, 73, 82], management [12, 57, 61, 66, 70, 77, 82], families [12, 26, 54, 65, 77, 82], service users [39, 47, 50, 61, 65], experts [40, 64] and evaluators [13, 14, 38, 44, 52, 58, 61, 70, 75, 77, 81]. Many used multiple methods, for example, Mookheriji and Lafond used immunisation programme theory and discussion with programme stakeholders, including immunisation experts, to develop a ToC of routine immunisation performance [64]. They used a case study approach to evaluate immunisation performance and then refined the ToC based on the results of this evaluation and a stakeholder workshop.

The resultant ToCs were described using narrative summaries (*n* = 15, 34.1 %), diagrams (*n* = 22, 50 %) or both (*n* = 6, 13.6 %). In one case, a table was used. Table 4 outlines the components of the ToCs that were described. Almost all of the ToCs outlined the long-term outcome required, and the majority described the process or sequence of change. However, assumptions and indicators were displayed or described infrequently.

**Table 4:** Components of ToC in the papers where a ToC was displayed or described. Essential and additional components adapted from Vogel [8]

ToC components	*n* = 44
	*n* (%)
Essential	
Long-term change	40 (90.9 %)
Process/sequence of change	33 (75 %)
Context	24 (54.5 %)
Assumptions	7 (15.9 %)
Additional	
Strategic choices and intervention options	23 (52.3 %)
Beneficiaries	20 (45.5 %)
Actors in the context	13 (29.5 %)
Timeline	4 (9.1 %)
Indicators	4 (9.1 %)
Sphere of influence	3 (6.8 %)

### Using ToCs to design public health interventions

Eighteen papers (29 %) described the use of ToC in the development of a public health intervention. The majority of these reported that they used the ToC as a framework for the intervention [12, 31, 42, 43, 70] or as a basis for a strategic plan [61, 68, 69, 76, 82]. Some examples of how ToCs were used to design public health interventions follow. Basson et al. used formative research to develop a ToC for a workplace wellness intervention for HIV-affected nurses and presented this programme theory as a framework for future research. Lund et al. used stakeholder workshops to develop their ToC and then used this to refine the substance and delivery of integrated district mental healthcare plans in five low- and middle-income countries [55]. A few presentations and papers reporting the development of systems of care for children with behavioural difficulties used the ToC as an outline of their public health intervention and as a basis for their strategic plan [12, 61, 65]. Chandani et al. [40] used the ToC to frame the results of their formative work and used the ToC to identify interventions to address the bottlenecks to the availability of essential medicines among community health workers in Ethiopia, Malawi and Rwanda.

### Using ToCs to evaluate public health interventions

Forty-nine papers (79 %) describe the use of ToC in the evaluation of the intervention. This includes the development of indicators, the overall evaluation design and data analysis.

The development of indicators used in the ToC was described in 28 papers. The indicators were often developed from the short-, medium- or long-term outcomes described in the ToC [27, 35, 38, 58, 65, 74, 81, 84]. Thirty-two (51.6 %) measured process constructs, 28 (45.2 %) measured outcome constructs and 9 (14.5 %) measured contextual constructs described in the ToC. Only two papers [12, 82] explicitly described the use of ToC to identify indicators for ongoing monitoring of the implementation of the intervention.

The majority of papers (62.9 %) reported formulating their evaluation questions around the ToC. However, the papers varied in the amount of detail they provided on this process. A common description was that the ToC was used to provide a framework for the evaluation [27, 32, 33, 48, 64, 72, 74, 78, 79, 81, 82]. Others reported that they used the evaluation to develop [39], refine [40] or validate the ToC [64]. Two papers reported that their evaluation was guided by testing the assumptions in the ToC [29, 34].

The data collection and analysis methods used varied greatly across papers. Data collected for the evaluation included routinely collected data [33, 44], custom-designed surveys [13, 16, 32, 72, 76, 79] and qualitative data. Qualitative data collection methods included interviews [13, 27, 35, 47, 71, 73, 75, 79], programme observation [13, 27, 35], programme documentation [13, 35, 71, 75, 79] and visual evidence [32]. The quantitative data analysis methods were strongly linked to the types of data collected and included descriptive statistics [33], inferential statistics [27, 40, 42, 43, 74, 78], multilevel modelling [16] and path analysis [41]. Other methods included case study approaches [16, 33, 36, 64] and iterative thematic analysis [71] whereas others did not explicitly state their specific data analysis approach [14, 77].

Few papers explicitly explored the influence of context of the intervention in relation to ToC. Although some ToCs mentioned context, particularly those with a realist evaluation focus, there was little description of how context affected the interpretation of the evaluation. There were some exceptions [40, 56, 64, 72]. Mookherji and LaFond used a case study approach to explore what worked within and between immunisation programme contexts to identify common factors influencing immunisation performance in Ghana, Ethiopia and Cameroon [64]. For example, political and social commitment to routine immunisation was seen as a key factor in influencing immunisation performance although it was described slightly differently for each context. Similarly, Chandani et al. developed a cross-country ToC of community health worker supplied medication in Ethiopia, Malawi and Rwanda. They compared whether each of the preconditions and the outcome was achieved in each setting [40]. These differences were then explained based on the contextual factors in each setting such as types of medication provided by the health workers, standard operating procedures and data availability and means of transport and travel times. Secker et al. [72] explored the influence of socioeconomic and demographic characteristics as well as infrastructure and organisational processes and systems between eight pilot sites in the evaluation of a preretirement health initiative.

### Using ToC to provide causal explanations

Few papers reported on the identification of breakdowns and side effects, effectiveness or efficacy and causal explanation as described by Coryn et al. [4]. Only four (6.5 %) identified breakdowns of programme theory, three (4.8 %) identified unexpected consequences of the intervention, ten (16.1 %) made cause-and-effect associations between theoretical constructs explicit, two (3.2 %) described differences in direction and/or strength of relationship between programme and outcomes and two (3.2 %) described the extent to which one construct accounted for/mediated the relationship between other constructs.

## Discussion

In this systematic review, we provide an overview of how ToCs have been developed and used to develop and evaluate public health interventions. As expected, there is variation in how ToCs are developed and used in evaluation although the papers report very little detail about the ToC process.

We have shown that the ToC approach has been in use since at least 1999 with 62 papers found in peer-reviewed journals and grey literature. This was significantly more than expected, given that Coryn et al. [4] found only three papers describing theory-driven evaluation of health interventions using ToC. However, Coryn et al. only included papers that reported the use of ToC for evaluation (rather than also describing the development of ToC or the use in the design of an intervention) and excluded those that did not provide enough detail [4].

In this review, many papers provided little detail in relation to the process of ToC development and how the ToC was used to design the intervention or conduct the evaluation. For example, Bonner [36] describes the ToC approach in detail but provides only a short example of the Health Action Zones experience of using ToC to evaluate an intervention to reduce drug taking. Brown et al. [37] reports using a ToC approach to evaluate a health promotion intervention for adolescents. The only description of ToC was found in the abstract and then mentioned briefly in the discussion. There was no clarity on how the ToC was developed or any explicit mention of how it was used to inform the analysis.

In contrast, other papers provided extensive detail on the ToC development process. For example, Hernandez and Hodges [12] describe the 12 step process used to develop a ToC for interagency delivery of mental health services for children with serious emotional disturbances and their families. They describe each step in detail including the purpose of the stage of the process, the types of stakeholders participating in the step, the substance of the discussions and the decisions reached. The ToC was then displayed as a logic model for readers to gain a better understanding of the output of the process. Similarly, Mookherji and LaFond [64] described in detail their approach to developing their initial ToC and how the ToC was used to determine case selection for a comparative case study. They then described how they used the results of the comparative case study and the ToC workshops to refine their ToC.

A range of methods were used to develop ToCs. The methods ranged from participatory methods which encourage stakeholder participation and ownership of the ToC such as workshops and working groups, to more evaluator focused approaches such as programme observation and review of programme documentation. Although the reason for the choice of methods was rarely made explicit by the authors, these methods were presumably chosen based on the purpose, depth and level of stakeholder buy-in the ToC required. For example, the examples of the development of systems of care for children and adolescents with mental and behavioural disorders viewed stakeholder participation as very important and therefore held a series of workshops with multiple stakeholders from different government departments, service providers, families and service users [12, 50, 61]. In some cases, although stakeholders were interviewed or participated in surveys, they did not contribute explicitly to the development of the ToC [16, 57]. Sullivan and Stewart [10] argue that although participation of all stakeholders in the development of ToC is the ideal presented by Weiss and colleagues [7], this is not always practical or feasible. They argue that different types of ToC development and resulting ownership may have advantages and therefore it is important to be explicit about the development process.

The lack of detail in most of the examples in this review makes it difficult to assess the thoroughness of ToC development. In many cases, the ToC seems to have been developed superficially and then used in a cursory way during evaluation. Similarly, where diagrams or narrative summaries of ToCs are presented in the papers, very little detail is included. Most authors present the long-term outcomes, sequence of change, beneficiaries and context. However, very few make their assumptions explicit although Vogel identifies these as a core part of ToC [8]. Where ToC was used to develop the interventions, it was often not clear how this was done apart from providing an overarching framework or strategic plan for the intervention.

A surprising finding of the review was the paucity of papers that describe the use of ToC for use during the implementation of the intervention (*n* = 2). Given the popularity of ToC as a monitoring and evaluation tool by international development agencies such as the Department for International Development, UK, [8], we had expected that more papers would use ToC during the implementation phase to assess progress towards the outcomes as well as modify implementation where necessary.

ToC theorists such as Connell and Kubisch [15] emphasise that the ToC approach to evaluation is method neutral and, as such, does not prescribe a specific type of study design or evaluation method. This was reflected in the papers included in this review which used a variety of qualitative and quantitative data collection and analysis methods. This flexibility in methods can be an advantage if researchers can design evaluations which seek to understand and evaluate both the outcomes and causal mechanisms which are made explicit in the ToC. However, flexibility in methods may also result in evaluations being poorly formulated in terms of the appropriateness of the methods, the rigor of data analysis or the results not interpreted in light of the ToC. In this review, evaluations were often described in detail but it was not clear how they linked to the ToC or how the ToC was used to interpret the results. However, some authors clearly develop or refine their ToCs as the results of the evaluation emerge. For example, Carroll et al. [39] sought to describe a theory of change for health promotion activities for hard to reach groups which was developed through the evaluation.

Most papers failed to explicitly discuss the results of the ToC in relation to unexpected outcomes, direction of causation and mediation of effects. This is similar to the conclusions drawn by Coryn et al. who report that programme theory was not used in any meaningful way to develop evaluation questions or plan and conduct and interpret the analysis [4].

It is interesting to note that no studies used ToC alongside RCTs as a method to unpack the programme theory underpinning the intervention. As we have noted previously, ToC holds much potential for this as RCTs alone are no longer considered adequate for the evaluation of complex health interventions [3].

Detailed reporting of the ToC process is particularly important as definitions of ToC differ considerably [8]. Many papers did not define ToC. However, there were clear overlaps with other theory-driven evaluation approaches, in particular, realist approaches [32, 36, 38, 56] and logic models [12, 26, 31, 52, 54, 61, 65, 66, 68, 69, 76, 82]. Realist approaches have a different theoretical basis to ToC and differ in several ways including how they articulate and generate theory, the degree to which stakeholders are involved and the types of knowledge they seek to generate [20]. Marchal et al. [18], in a systematic review on realist evaluation in health systems research, also noted that ToC and realist evaluation were often used together or interchangeably. Logic models are conceptually similar to ToC but are usually presented in a linear form with boxes for inputs, activities, outputs and outcomes with little explanation of the causal pathways linking them [3]. Reducing a ToC to a logic model may conceal some of the explanatory power of the causal pathways.

Two limitations to this review are the lack of double data extraction and the inability to effectively measure the quality of the included papers. We did extract data on a checklist of ToC components proposed by Vogel [8] and principles of theory-driven evaluation by Coryn et al. [4], but it was difficult to make an assessment of quality. This is primarily because there is no agreed upon quality criteria for ToC. This is compounded by the flexibility of the ToC approach, both in the development of ToCs and how they can be used for evaluation. Because evaluations using ToC vary in study design and method, existing methodological checklists are of little use for comparative purposes.

We suggest that authors planning to report on ToC to guide the development or evaluation of public health interventions provide more detail on the ToC process to readers. In particular, it is important to make the ToC used explicit and this is usually easier in diagrammatic form. Complex ToCs can be simplified in a summary diagram with detailed ToCs provided as web appendices. This will help the reader to understand the authors’ expected pathways of change and judge their validity. In addition, it is imperative that authors describe in detail how the ToC was developed and used. This is particularly important as there is no single way to develop or use a ToC. Making the process explicit helps readers judge the credibility of the ToC and strengthen the literature in this field.

We have therefore developed a checklist based on this review and the work of Coryn et al. [4] and Vogel [8] which can assist with the clearer reporting of the ToC approach. The checklist gives guidance as to which aspects of the ToC should be made explicit (Table 5). It covers five domains, namely the (1) definition of ToC; (2) description of the ToC development process; (3) ToC diagram; (4) process of intervention development and (5) use of ToC in evaluation. The checklist would benefit from expert review and piloting in the real world. However, it provides a starting point for authors reporting a ToC approach. As ToC is method neutral, this checklist could also be used together with other existing checklists such as the CONSORT statement for RCTs [85], the STROBE guidelines for observational research [86] or CReDECI2 for complex intervention development and evaluation [87].

**Table 5:** Checklist for reporting ToC in Public Health Interventions

1. Is the ToC approach defined?	
	a. Is a definition of ToC given by the authors?
	b. Do the authors explain their reasons for using a ToC approach?
2. Is the ToC development process described?	
	a. Are the methods used to develop the ToC, such as stakeholder meetings and interviews, document reviews, programme observation, existing conceptual frameworks or published research, described?
	b. Where stakeholders are involved, is it clear how many stakeholders participated, what their role is in relation to the intervention, how they were consulted (e.g. number of interviews, focus groups, ToC workshops) and the extent to which the consultations were participatory?
	c. Is the method used to compile the data into a ToC described? (including how disagreements between stakeholders were resolved)
	d. Is the extent to which stakeholders were able to validate the resultant ToC and were owners of the final product described?
3. Is the resultant ToC (or a summary thereof) depicted in a diagrammatic form and does it include?	
	a. The long-term outcome or impact of the intervention
	b. The anticipated short and medium term outcomes and the process of change
	c. The intervention components which happen at different stages of the pathway
	d. The context of the intervention
	e. Assumptions about how change would occur
	f. Additional ToC elements such as indicators, supporting research evidence, beneficiaries, actors in the context, sphere of influence and timelines where relevant.
4. Is the process of intervention development from the ToC described?	
	a. Are the methods of how interventions were refined from the ToC to something which can be implemented described? (For example, further stakeholder workshops, interviews, systematic literature reviews)
5. Is the way in which the ToC was used to develop and implement the evaluation described?	
	a. Are evaluation research questions generated from the ToC?
	b. Is the role of ToC in the design, plan or conduct of the evaluation clear?
	c. Does the paper describe the extent to which the key elements described in the ToC were measured in the evaluation (i.e. impact, short and medium term outcomes and the process of change, context, assumptions and the intervention)?
	d. Does the paper describe whether and how process indicators were used to improve the quality of the intervention?
	e. Is the role of the ToC in the analysis of the results of the evaluation clear?
	f. Is the role of ToC in the interpretation of the results of the evaluation described? (including the breakdown of programme theory, unanticipated outcomes and causation including the strength and direction of causal relationships)

## Conclusion

The ToC approach is widespread in the public health literature. Clear reporting of the ToC process and outputs is important to improve to allow the readers a thorough understanding of the work and allows them to judge the validity of the approach. We recommend that our proposed checklist is used and refined by authors reporting the ToC approach.

## Additional file

Additional file 1: The use of Theory of Change to design, implement and evaluate Public Health Interventions: a systematic review protocol. (DOCX 202 kb)

## Abbreviations

RCT: randomised controlled trial
ToC: theory of change
