# Supplementary material for: Using theory of change to design and evaluate public health interventions: a systematic review
Source: Implement Sci. 2016 May 6;11:63. doi: 10.1186/s13012-016-0422-6 (PMC4859947; doi:10.1186/s13012-016-0422-6)
Supplement: Additional file 1: — The use of Theory of Change to design, implement and evaluate Public Health Interventions: a systematic review protocol. (DOCX 202 kb) [file 13012_2016_422_MOESM1_ESM.docx]

The use of Theory of Change to design, implement and evaluate Public Health Interventions: a systematic review protocol

Erica Breuer, Lucy Lee, Mary De Silva, Crick Lund

Contents

[2 Aim and research questions 2](#_Toc372794075)

[3 Operational Definitions 3](#_Toc372794076)

[4 Data collection and tools 4](#_Toc372794077)

[5 Data analysis 7](#_Toc372794078)

[6 Credibility and transferability 7](#_Toc372794079)

[Appendix: Data Collection Forms for the Systematic Review 8](#_Toc372794080)

[i. Systematic Review Database Search Log 8](#_Toc372794081)

[ii. Systematic Review Abstract and Full Text Screening Criteria 9](#_Toc372794082)

[iii. Systematic Review Quality Assessment form for Theory-Driven Evaluation Papers 10](#_Toc372794083)

[iv. Systematic Review Quality Assessment form for core elements of ToC 11](#_Toc372794084)

[v. Systematic Review Data Extraction Form 13](#_Toc372794085)

# Aim and research questions

Aim

To explore and critically evaluate the literature on ToC and its use in the design, implementation and evaluation of PHIs

Research Questions

A systematic review will be used to identify, compare and evaluate studies which have used ToC for the design, implementation and/or evaluation of public health interventions (PHIs) to answer the following questions:

1. How do papers using ToC define ToC?
2. Do what extent do the papers who self-identify as using ToC report using the principles of Theory Driven Evaluation as identified by [Coryn, Noakes et al. (2011)](#_ENREF_1) and the attributes of ToC identified by [Vogel (2012)](#_ENREF_5).
3. How are programme theories for PHIs developed and refined in ToC approaches?
4. How does the ToC approach and the explicated programme theory influence the
   1. Development of intervention;
   2. Implementation of the intervention;
   3. Development of indicators for measurement;
   4. Evaluation of the intervention, including statistical approaches;
   5. Conceptualisation/evaluation of influence of context; and
   6. Causal attribution of the intervention?

# Operational Definitions

Theory of Change (ToC): an outcomes-based approach which describes how an intervention brings about specific outcomes through a logical sequence of intermediate outcomes ([Vogel 2012](#_ENREF_5)). ToC is distinct from sociological or psychological theories which describe why change occurs although these may be used to inform the ToC ([De Silva, Breuer et al. (in preparation)](#_ENREF_2))

Interventions: *“a set of actions with a coherent objective to bring about change or produce identifiable outcomes. These actions may include policy, regulatory initiatives, single strategy projects or multi-component programmes.”*  ([Rychetnik, Frommer et al. 2002](#_ENREF_4))

Public Health Interventions (PHI): “*interventions [which] are intended to promote or protect health or prevent ill health in communities or populations. They are distinguished from clinical interventions, which are intended to prevent or treat illness in individuals.”* ([Rychetnik, Frommer et al. 2002](#_ENREF_4))

The principles of Theory Driven Evaluation as identified by Coryn, Noakes et al. (2011) and the attributes of ToC identified by [Vogel (2012)](#_ENREF_5) are outlined in Appendix iii and iv.

# Data collection and tools

The systematic literature review will involve searching of the key databases of both peer-reviewed journal articles and other research outputs listed in Box 1, Figure 1.

*Databases of Peer-Reviewed Journal Articles*

- Scopus
- Pubmed
- PsychInfo
- Science Citation Index
- Social Science Citation Index
- Academic Search Premier
- Africa-Wide Information
- CINAHL
- BIOSIS

*Databases of other research outputs (Grey Literature)*

- The Directory of Published Proceedings
- Google (first 50 pages)
- OpenGrey
- PsycEXTRA
- Disability Archive UK
- Eldis
- Popline
- DFID Research for Development
- SciDevNet
- World Bank Documents and Reports

The websites of Comic Relief, DFID, Grand Challenges Canada, The Bill and Melinda Gates Foundation, HIVOS, World Vision, the Robert Wood Johnson foundation, Acktknowledge, and the Theory of Change Community will also be searched.

The search process will be recorded using a Database Search Log (Appendix i). As the purpose of the systematic review is to identify only those articles who report using ToC, I will search only for theory of change in all fields of the document. For databases which are not specific to healthcare I will also add the following search terms: health, health care, health services, and medicine.

To test the sensitivity of this search strategy, the search results will be checked for articles already identified from a previous systematic review of TDE by [Coryn, Noakes et al. (2011)](#_ENREF_1). The review by Coryn et al. was much broader in scope than the proposed review and included any type of program evaluation in any discipline. It focused on whether studies using TDE adhered to the principles of TDE. Our study will investigate only ToC and how this has been used in the design, implementation and evaluation of public health interventions specifically. We will also investigate how the ToC approach was used in each stage of the programme development and evaluation.

In addition, reference sections of retrieved articles will be checked for further references and a citation analysis of key papers performed using the Scopus, Science Citation Index and Social Science Citation Index. We will also include papers found through contacting experts in the field (Greenhalgh and Peacock, 2005).

Following the search, the titles and abstracts of the search results will exported into Endnote ([Endnote 2011](#_ENREF_3)) where duplicates and irrelevant titles will be removed. Then the titles and abstracts will be screened by two reviewers, EB and LL using the Systematic Review Abstract and Full Text Screening Criteria (Appendix II).

Once the abstracts are screened for inclusion and exclusion criteria, the full papers will be obtained and assessed for eligibility by both reviewers. Papers that meet the inclusion and exclusion criteria will be included in the review and checked against the criteria proposed by [Coryn, Noakes et al. (2011)](#_ENREF_1) for assessing papers reporting TDE and the components of ToC proposed by [Vogel (2012)](#_ENREF_5) (Appendix iii). Studies will not be excluded on the basis of quality, but study quality will be used to assess possible heterogeneity in the results. Any differences between authors throughout the review process will be resolved via discussion.

The data from the content of the paper will be extracted by the first author (EB) using the attached form (Appendix v).


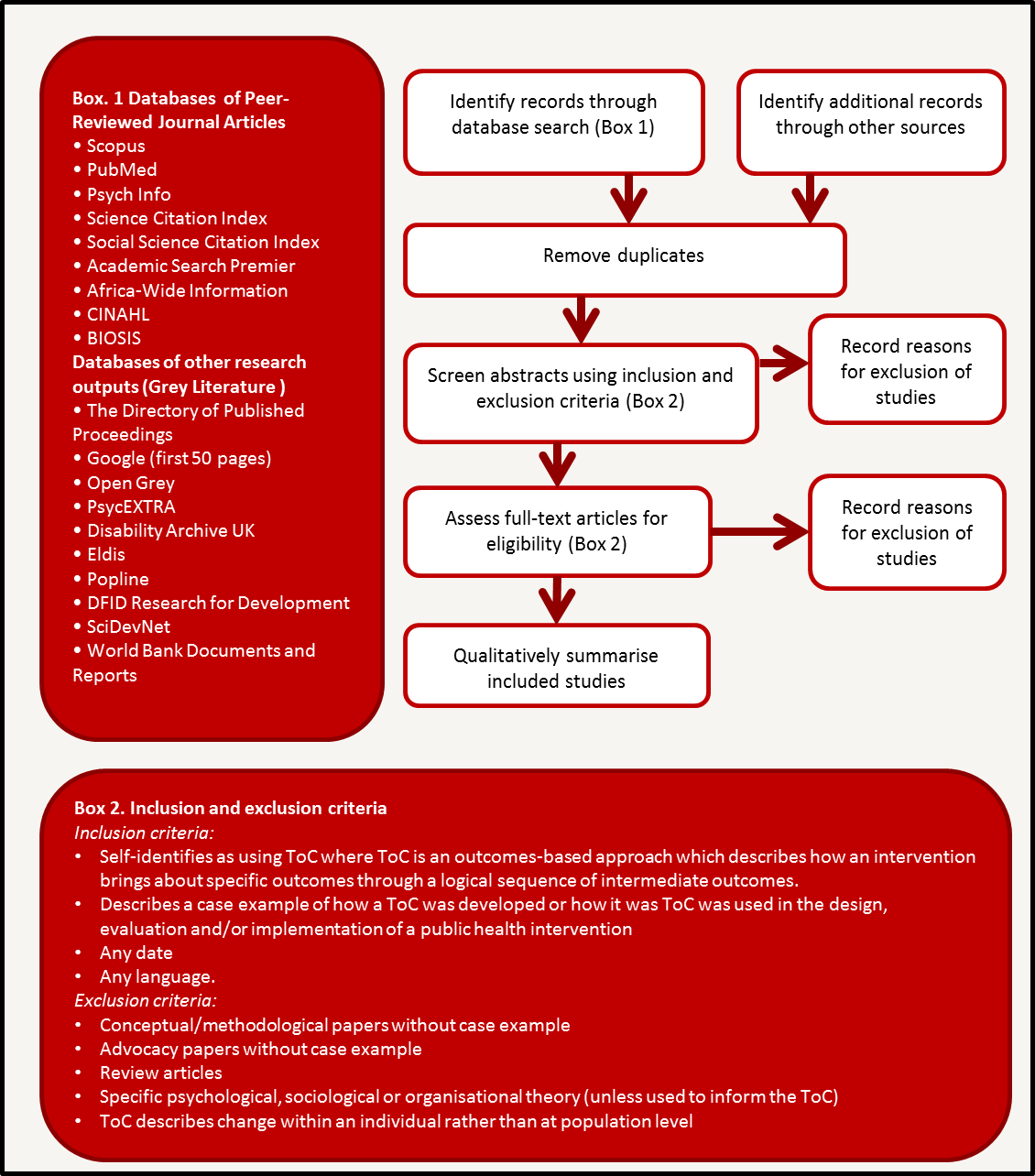


Figure 1 The Stages of the Systematic Literature Review

# Data analysis

Once the data is extracted, studies will be compared, evaluated and summarised qualitatively in relation to the research questions described above. Key lessons regarding the use of ToC for designing, implementing and evaluating PHIs will be synthesised.

# Credibility and transferability

This review will employ various strategies to ensure that it is an unbiased representation of the literature. Firstly, a thorough *a priori* search strategy will be used to find relevant studies. We will include several databases specifically indexing grey literature in order to locate studies which are not reported in peer-reviewed journals. We will ensure our search strategy is adequate by checking the results against known articles which are relevant and by contacting experts in the field. Lastly, all potential abstracts and full text reports will be screened by two reviewers who will determine whether these studies should be included or excluded in the review.

# Appendix: Data Collection Forms for the Systematic Review

## Systematic Review Database Search Log

| Database | Date | Search Strategy | No. of hits | Comments |
| --- | --- | --- | --- | --- |
|  |  |  |  |  |
|  |  |  |  |  |
|  |  |  |  |  |
|  |  |  |  |  |
|  |  |  |  |  |
|  |  |  |  |  |
|  |  |  |  |  |
|  |  |  |  |  |
|  |  |  |  |  |
|  |  |  |  |  |
|  |  |  |  |  |
|  |  |  |  |  |

## ii. Systematic Review Abstract and Full Text Screening Criteria

| Topic | Criteria | Yes | Unclear | No |
| --- | --- | --- | --- | --- |
| Review article | Is this a review article? |  |  |  |
| Public Health | Public Health intervention |  |  |  |
| ToC | Self-identifies as using ToC where ToC is an outcomes-based approach which describes how an intervention brings about specific outcomes through a logical sequence of intermediate outcomes. |  |  |  |
|  | The ToC is not a specific organisational, sociological or psychological theory only (although the ToC may be based on one) but describes the public health intervention in question |  |  |  |
|  | The ToC explores changes within the population rather than within individuals |  |  |  |
|  | Uses ToC in the design, evaluation and/or implementation of an intervention |  |  |  |
| Advocacy/methodological paper only | Does it include a case example? |  |  |  |

Instructions

Abstract screening

- Find Full Text if Review (No/Unclear) AND TOC (Yes/Unclear to all) AND Public Health (Yes/Unclear)
- Exclude if Review (Yes) OR TOC (No to any) OR Public Health (No)
- Exclude if advocacy/methodological paper with no case example

Full text screening

- Include full text in review if review (No) AND TOC (Yes to all) AND Public Health (Yes)
- Exclude if Review (Yes) OR TOC (No to any) OR Public Health (No)
- Exclude if advocacy/methodological paper with no case example

For full text screening the order of the inclusion and exclusion criteria will be changed to increase efficiency. The criteria remained the same.

| Topic | Criteria | Yes | Unclear | No |
| --- | --- | --- | --- | --- |
| ToC | Self-identifies as using ToC where ToC is an outcomes-based approach which describes how an intervention brings about specific outcomes through a logical sequence of intermediate outcomes. |  |  |  |
|  | Describes a case example of how a ToC was developed or how it was ToC was used in the design, evaluation and/or implementation of a public health intervention |  |  |  |
| Advocacy/methodological paper only | Does it include a case example? |  |  |  |
| Review article | Is this a review article? |  |  |  |
|  | The ToC is not a specific organisational, sociological or psychological theory only (although the ToC may be based on one) but describes the public health intervention in question |  |  |  |
|  | The ToC explores changes within the population rather than within individuals |  |  |  |

## Systematic Review Quality Assessment form for Theory-Driven Evaluation Papers

| Criteria  (Adapted from [Coryn, Noakes et al. (2011)](#_ENREF_1)) | | Yes | Page # | No | Not enough detail provided | Comments |
| --- | --- | --- | --- | --- | --- | --- |
| A | **Theory formulation** |  |  |  |  |  |
|  | **Programme theory is formulated** | | | | | |
| A1 | from existing theory and research |  |  |  |  |  |
| A2 | from implicit theory |  |  |  |  |  |
| A3 | from observation of the program in operation/exploratory research |  |  |  |  |  |
| B | **Theory-guided question formulation and prioritisation** |  |  |  |  |  |
| B1 | Evaluation questions were formulated around program theory |  |  |  |  |  |
| B2 | Evaluation questions were prioritised | Yes (Go to B3) |  | No (Go to B4) |  |  |
| B3 | Evaluation questions are prioritised according to: | Funding priorities | | Logistical constraints | Programme theory |  |
|  |  | Other | | Describe: | |  |
| C | **Theory-guided planning, design, and execution** | | | | | |
| C1 | A programme theory is used to design, plan and conduct evaluation |  |  |  |  |  |
| C2 | Programme theory is used to determine whether the evaluation was designed considering relevant contingencies |  |  |  |  |  |
| C3 | Programme theory was used to determine whether the evaluation should be tailored or comprehensive |  |  |  |  |  |
| D | **Theory-guided construct measurement** | | | | | |
| D1 | Process constructs postulated in programme theory are measured |  |  |  |  |  |
| D2 | Outcome constructs postulated in programme theory are measured |  |  |  |  |  |
| D3 | Contextual constructs postulated in programme theory are measured |  |  |  |  |  |
| E | **Identification of breakdowns and side effects, effectiveness or efficacy, and causal explanation** | | | | |  |
| E1 | Breakdowns of programme theory are identified |  |  |  |  |  |
| E2 | Outcomes were identified which were NOT postulated by program theory |  |  |  |  |  |
| E3 | Cause-and-effect associations between theoretical constructs are described |  |  |  |  |  |
| E4 | Cause-and-effect associations between theoretical constructs are explained |  |  |  |  |  |
| E5 | Differences in direction and/or strength of relationship between program and outcomes are explained |  |  |  |  |  |
| E6 | The extent to which one construct accounts for/mediates the relationship between other constructs is explained |  |  |  |  |  |
|  |  |  |  |  |  |  |

## Systematic Review Quality Assessment form for core elements of ToC

| Core elements of a ToC (Adapted from [Vogel (2012)](#_ENREF_5)) | | Yes/No | Page No |
| --- | --- | --- | --- |
| Context | for the initiative, including social, political and environmental conditions and other actors able to influence change |  |  |
| Long-term change | that the initiative seeks to support and for whose ultimate benefit |  |  |
| Process/sequence of change | that is anticipated in order to create the conditions for the desired long-term outcome |  |  |
| Assumptions | about how these changes might happen, as a check on whether the activities and outputs are appropriate for influencing change in the desired direction in this context. |  |  |
| Diagram and narrative summary | that captures the outcomes of the discussion. |  |  |
| Additional Components | |  |  |
| Beneficiaries | Focus on who is intended to benefit from the changes in the context that the programme or intervention aims to support, and how they will benefit. |  |  |
| Actors in the context | Analysis of the actors, organisations and networks that influence change in the setting, power relationships and institutional configurations |  |  |
| Sphere of influence | Analysis of the programme’s, ability to reach and influence change, directly through its interventions or indirectly through collaboration and interaction |  |  |
| Strategic choices and intervention options | Activities needed to influence the changes sought |  |  |
| Timeline | A realistic timeframe for changes to unfold, expected trajectory of changes following interventions |  |  |
| Indicators | Areas to investigate and track with evaluation and impact assessment. |  |  |

## Systematic Review Data Extraction Form

| Variable | Response |
| --- | --- |
| Reference | [text] |
| Year | [number] |
| Language | [text] |
| Location(s) | [text] |
| Type of publication | [Grey (1) /Postgrad Dissertation (2)/Published- peer reviewed (3)] |
| Journal | [text] |
| Journal Field | [text] |
| Brief description of programme | [text] |
| Integrated into routine health services | [yes/no] |
| Mental Neurological and Substance Use intervention | [yes/no] |
| Set in low and middle income country | [yes/no] |
| Type of health intervention | [text] |
| Health outcome | [text] |
| Other outcomes | [text] |
| Describes development of TOC | [yes/no] |
| How was the TOC developed? | [text] |
| Retrospective or prospective theory development | [retrospective/prospective/neither] |
| How are TOC for PHIs refined? | [text] |
| Describes Role of ToC in development of intervention | [yes/no] |
| Role of TOC in: The development of the intervention | [text] |
| Describes role of ToC in the implementation of the intervention | [yes/no] |
| Role of TOC in: Implementation of the intervention | [text] |
| Describes the Role of ToC in the evaluation of the intervention | [yes/no] |
| Role of TOC in: Development of indicators for measurement | [text] |
| Role of TOC in: Evaluation of the intervention, including statistical approaches | [text] |
| More detail on statistical analysis | [text] |
| Role of TOC in:Influence of context | [text] |
| Role of TOC in: Causal attribution of the intervention | [text] |
| Role of TOC in generalising the results to other programmes | [text] |
| Potential Biases or concerns | [text] |
| Other points or key lessons for reflection from the authors | [text] |
| Notes | [text] |

1. Coryn, C. L. S., L. a. Noakes, C. D. Westine and D. C. Schroter (2011). "A Systematic Review of Theory-Driven Evaluation Practice From 1990 to 2009." American Journal of Evaluation **32**: 199-226.
2. De Silva, M., E. Breuer, L. Lee, C. Lund and V. Patel ((in preparation)). "Theory of change: a theory-driven approach to the MRC framework for complex interventions ".
3. Endnote (2011). San Fransisco, Thomson Reuters.
4. Rychetnik, L., M. Frommer, P. Hawe and A. Shiell (2002). "Criteria for evaluating evidence on public health interventions." Journal Of Epidemiology And Community Health **56**: 119-127.
5. Vogel, I. (2012). Review of the use of ‘Theory of Change’ in international development. UK, Department for International Development (DFID).
